# Development of a liver stiffness measurement-based nomogram model to identify early CKD risk in patients with MAFLD

**DOI:** 10.1186/s12876-026-04701-z

**Published:** 2026-03-06

**Authors:** Dejin Huang, Xiaoxuan Ma, Qingchen Gao, Xiaolong Shen, Tongtong Yu, Rongqi Wang

**Affiliations:** Department of Traditional and Western Medical Hepatology, Hebei Provincial Key Laboratory of Liver Fibrosis in Chronic Liver Diseases, Hebei Medical University Third Hospital, Shijiazhuang, China

**Keywords:** Metabolic-associated fatty liver disease, Chronic kidney disease, Nomogram model, Transient elastography

## Abstract

**Objective:**

To identify independent relevant factors for chronic kidney disease (CKD) in patients with metabolic dysfunction-associated fatty liver disease (MAFLD) and to develop and validate a nomogram-based risk diagnostic model for MAFLD-CKD incorporating liver stiffness measurement (LSM).

**Methods:**

Clinical data from 3,154 patients with fatty liver disease attending our hospital between January 2024 and September 2025 were collected. According to the 2024 guidelines for the prevention and treatment of metabolic dysfunction-associated (non-alcoholic) fatty liver disease, a total of 1,328 MAFLD patients were ultimately included. Body mass index (BMI) was calculated, and controlled attenuation parameter (CAP) and LSM were measured using transient elastography (TE). Logistic regression analysis was employed to screen for independent relevant factors identifying MAFLD-CKD risk, which were used to construct a nomogram model. The study subjects were randomly divided into a training set (*n* = 930) and a validation set (*n* = 398) at a 7:3 ratio for internal validation of the model’s feasibility. The model performance was evaluated using the area under the receiver operating characteristic (ROC) curve (AUC) and the Hosmer-Lemeshow goodness-of-fit test.

**Results:**

Age, LSM, ALT, AST, total cholesterol (TC), triglycerides (TG), diabetes mellitus (DM), and fasting plasma glucose (FPG) were significantly higher in the MAFLD-CKD group compared to the MAFLD-only group. Multivariate logistic regression revealed that Age, LSM, TC, and the presence of DM were independent relevant factors for CKD in MAFLD patients (all *P* < 0.01). The diagnostic model combining Age, LSM, TC, and DM status achieved an AUC of 0.899 (95% CI: 0.882–0.917) for early identification of MAFLD-CKD, with a sensitivity of 0.71 and a specificity of 0.80, significantly outperforming any single indicator. A nomogram diagnostic model was successfully developed based on these variables. In the diagnostic model, the AUC for identifying MAFLD-CKD occurrence was 0.91 (95% CI: 0.89–0.93) in the training set and 0.88 (95% CI: 0.85–0.92) in the validation set. The Hosmer-Lemeshow test indicated no statistically significant difference between the training and validation sets (*P* > 0.05).

**Conclusion:**

Age, LSM, TC, and the presence of DM are independent factors associated with CKD in patients with MAFLD. The risk assessment model integrating these factors significantly improves the ability to differentiate MAFLD patients with existing early-stage CKD risk, demonstrating good discriminatory performance. As a non-invasive marker of liver fibrosis, LSM can serve as a practical clinical indicator for identifying the subgroup of MAFLD patients associated with an elevated risk of CKD.

Non-alcoholic fatty liver disease (NAFLD) is the most common chronic liver disease worldwide. Its prevalence has risen in parallel with the obesity epidemic, affecting approximately 25% to 30% of adults globally [1–4]. With the deepening understanding of disease mechanisms, an international expert consensus in 2020 proposed renaming NAFLD to MAFLD [5]. MAFLD is not only associated with the development and progression of liver cirrhosis and hepatocellular carcinoma, but recent cohort studies have revealed a significantly increased comorbidity risk with CKD. A retrospective study involving 120,000 Asian individuals showed that the incidence of CKD in MAFLD patients was 1.8 times higher than in non-MAFLD individuals (HR = 1.83, 95% CI: 1.62–2.07), and it confirmed a faster annual decline rate in the estimated glomerular filtration rate (eGFR) among MAFLD patients [6, 7]. However, the pathological mechanisms underlying MAFLD-CKD occurrence have not been fully elucidated, and there is a lack of precise and convenient non-invasive early warning biomarkers for this comorbidity.

Hepatic fibrosis is a central driver in the progression of MAFLD. As a key quantitative indicator for assessing liver tissue elasticity, LSM has been demonstrated by multiple studies to be closely associated with hepatitis and the degree of fibrosis [8, 9]. Furthermore, hepatic fibrosis may promote systemic metabolic disturbances and a low-grade inflammatory state through the “liver-kidney axis,” yet the specific discriminative efficacy of LSM for MAFLD-related CKD still requires validation. This study systematically evaluates the discriminative performance of LSM in MAFLD-associated CKD and constructs a comprehensive assessment model that integrates liver fibrosis indicators with metabolic parameters. This effort aims to translate the “liver-kidney axis” mechanism into a clinical risk assessment tool, providing a more accurate and integrated predictive strategy for early kidney injury identification in MAFLD patients. Therefore, the objectives of this study are: (1) to identify independent predictors of CKD in MAFLD patients based on existing data; and (2) to develop an integrated diagnostic model combining hepatic fibrosis and metabolic indicators, thereby optimizing early kidney risk assessment strategies in MAFLD patients.

## Materials and methods

### Study participants

This study retrospectively selected 3,154 patients who visited the Department of Integrated Traditional Chinese and Western Medicine Hepatology at the Third Hospital of Hebei Medical University between January 2024 and September 2025 as the analysis cohort. According to the clinical criteria outlined in the 2024 “Guidelines for the Prevention and Treatment of Metabolic Dysfunction-Associated Fatty Liver Disease (2024 Edition)” [10], 2,814 patients met the inclusion criteria: diagnosis of fatty liver by imaging and/or histopathological evidence of ≥ 5% macrovesicular steatosis, along with at least one metabolic cardiovascular disease risk factor.

**Fig. 1:**
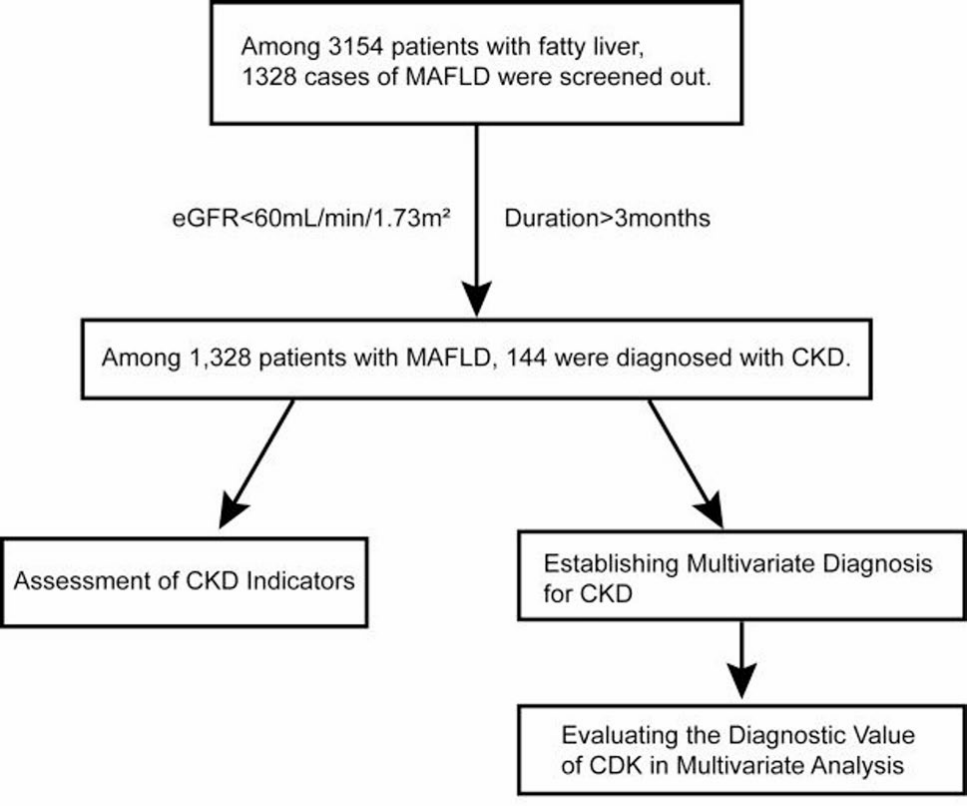
MAFLD patient screening process

Based on the following exclusion criteria: (1) Age < 18 years; (2) Weekly alcohol consumption > 140 g for males or > 70 g for females; (3) Fatty liver disease caused by other identified etiologies (e.g., alcoholic liver disease, viral hepatitis, autoimmune liver disease, drug-induced liver injury, etc.);(4) History of any malignant tumor; (5) Pre-existing CKD or significant renal impairment at baseline; (6) Current use of urate-lowering drugs (e.g., allopurinol, febuxostat, benzbromarone, etc.) prior to study enrollment, to avoid potential confounding effects of these medications on serum uric acid levels, renal function, or metabolic parameters of interest; (7) Missing key information, a total of 1,486 patients were excluded. Ultimately, 1,328 patients with MAFLD were included in the study (Fig. 1).

 A total of 1,328 patients with MAFLD were enrolled in this study. Based on their eGFR, they were divided into two groups: the MAFLD without CKD group (*n* = 1,184, eGFR > 60 mL/min/1.73 m²) and the MAFLD with CKD group (*n* = 144, eGFR < 60 mL/min/1.73 m² for more than 3 months).

According to the Guidelines for Early Screening, Diagnosis, and Prevention of Chronic Kidney Disease (2022 Edition) [11], chronic kidney disease is defined as an eGFR < 60 mL/min/1.73 m² persisting for more than 3 months, or a urine albumin-to-creatinine ratio (UACR) ≥ 30 mg/g. Given the retrospective design of this study, we defined the ‘chronic kidney disease group’ based on the following principles: (1) All patients met the criteria of eGFR < 60 mL/min/1.73 m² and/or UACR ≥ 30 mg/g based on data from this cross-sectional visit; (2) To align as closely as possible with clinical persistence, we further reviewed electronic medical records to ensure that patients in this group with reduced eGFR (< 60 mL/min/1.73 m²) had documented abnormal eGFR (also < 60 mL/min/1.73 m²) for at least 3 months prior to this visit, confirming that their eGFR decline was neither acute nor transient.

### Post-hoc power

To evaluate the diagnostic performance of a non-invasive method for detecting CKD, this study used the AUC as the primary indicator for sample size estimation. Under the conditions of a one-sided type Ⅰ error rate set at 0.05 and a statistical power of 90%, with an assumed CKD prevalence of 10% and a pre-specified AUC under the null hypothesis of 0.85, the minimum sample size required to test this hypothesis was calculated to be 1,089 subjects. During actual enrollment, the final sample size was expanded to 1,328 subjects. This sample size provides sufficient statistical power for the study based on the anticipated effect size.

### Study methods

#### Data collection

1. Basic Patient Information: Demographic characteristics, lifestyle behaviors, medication history, and medical history.
2. Physical Examination: Height: After removing shoes and hats, stand with the back against the column, ensuring that the heels, buttocks, shoulders, and occiput are in contact with the column. Look straight ahead. Gently press the headboard against the top of the head, and read the measurement to the nearest 0.1 cm. Perform two measurements and take the average.
  Weight: Wear lightweight clothing and remove shoes. Stand steadily at the center of the scale. After the body stabilizes, read the measurement to the nearest 0.1 kg. Perform two measurements and take the average.
  Blood Pressure: Use an upper-arm electronic blood pressure monitor. Measure after resting quietly for 5 minutes. Sit with the back supported, expose the upper arm, and position it at heart level. Secure the cuff properly. Measure three consecutive times with one-minute intervals between measurements, and record the average value.
3. Liver Biochemistry: 4 ml of fasting blood was collected in the morning, centrifuged, and analyzed using an Olympus AU2700 automatic biochemical analyzer to measure: Triglycerides (TG), Total Cholesterol (TC), High-Density Lipoprotein Cholesterol (HDL-C), Low-Density Lipoprotein Cholesterol (LDL-C), Fasting Plasma Glucose (FPG), Alanine Aminotransferase (ALT), Aspartate Aminotransferase (AST), and Blood Urea Nitrogen (BUN).
4. Renal Function: The estimated Glomerular Filtration Rate (eGFR) was calculated using the CKD-EPI 2021 combined creatinine-cystatin C formula [12].
5. Imaging Examination: LSM was performed using the FibroTouch transient elastography device (FT5000, manufactured by Wuxi Hisky Medical Technologies Co., Ltd.), with results expressed in kilopascals (kPa). Patients were examined in a fasting state while lying supine. The median of 10 valid measurements was used for analysis. Only cases with a success rate >60% and an interquartile range/median ratio <0.3 were included. All examinations were performed by the same professionally trained physician.

### Diagnostic criteria and definitions

Body Mass Index (BMI) was calculated as weight (kg) divided by height squared (m²) [13].

Dyslipidemia was diagnosed according to the *Chinese Guidelines for Lipid Management (Primary Care Edition*,* 2024)* [14], defined as meeting any one of the following criteria: TC ≥ 6.2 mmol/L, TG ≥ 2.3 mmol/L, LDL-C ≥ 4.1 mmol/L, or HDL-C < 1.0 mmol/L.

Diagnostic Criteria for Diabetes Mellitus: Glycated hemoglobin (HbA1c) ≥ 6.5%, fasting plasma glucose (FPG) ≥ 7.0 mmol/L (126 mg/dL), 2-hour plasma glucose during an oral glucose tolerance test (OGTT-2hPG) ≥ 11.1 mmol/L (200 mg/dL), or random plasma glucose (RPG) ≥ 11.1 mmol/L (200 mg/dL) with classic symptoms of hyperglycemia or hyperglycemic crisis [15].

### Statistical analysis

Statistical analysis was performed using SPSS 22.0 software. Measurement data conforming to a normal distribution are presented as mean ± standard deviation (x̄ ± s), while variables with a non-normal distribution are expressed as median (interquartile range). Comparisons between groups for normally distributed data were conducted using independent samples t-tests, and for non-normally distributed variables, the Mann-Whitney U test was applied. Count data are expressed as frequency (percentage), and differences between groups were assessed using the chi-square (χ²) test. Multivariate logistic regression analysis was employed to explore the associations between various indicators and their combined diagnostic value for MAFLD with CKD. Receiver operating characteristic (ROC) curves were plotted, and the area under the curve (AUC), sensitivity, and specificity were calculated to evaluate the diagnostic performance of each indicator for MAFLD complicated by CKD. A nomogram diagnostic model for the risk of MAFLD-CKD onset was constructed using the R 4.2.1 software package and the rms package. The discriminative ability of the model was analyzed through decision curve analysis (DCA). The Hosmer-Lemeshow test was used to assess the consistency between identification probabilities and actual observed values, reflecting model calibration through goodness-of-fit testing. Internal validation of the model was performed using the Bootstrap resampling method to enhance result reliability. A *P*-value < 0.05 was considered statistically significant.

## Results

### Comparison of clinical characteristics between MAFLD patients with and without CKD

This study included a total of 1,328 patients with MAFLD, among whom 144 (10.8%) had comorbid CKD. Compared to MAFLD patients without CKD, those with CKD were older, exhibited more pronounced abnormalities in glucose and lipid metabolism (elevated FPG, TG, and TC, *P* < 0.001), and showed differences in ALT and AST suggesting more severe liver injury, along with higher LSM. No significant differences were observed between the two groups in terms of gender, smoking proportion, blood pressure, CAP, HDL, LDL, or UA (Table 1).

**Table 1:** Baseline characteristics of patients with MAFLD

Variables	All patients (*n* = 1328)	MAFLD(*n* = 1184)	MAFLD and CKD(*n* = 144)	*P*
Age (years)	41.25 ± 15.33	39.4 ± 14.90	56.2 ± 9.60	0.038 *
Male [*n*(%)]	687(51.7%)	607(52.6%)	80(55.6%)	0.33
Smoker [*n*(%)]	264(19.9%)	233(19.7%)	31(21.5%)	0.60
DM[n(%)]	734(55.2%)	612(51.7%)	122(84.7%)	< 0.001***
SBP(mmHg)	136.75 ± 12.04	136.74 ± 12.10	136.8 ± 11.51	0.378
DBP(mmHg)	84.90 ± 8.66	85.0 ± 8.70	84.4 ± 8.60	0.169
BMI(kg/m²)	25.68 ± 2.65	25.34 ± 2.57	28.48 ± 1.45	0.182
CAP(dB/m)	259.6 ± 35.49	255.8 ± 35.43	290.6 ± 14.65	0.408
LSM(kPa)	7.92 ± 2.65	7.61 ± 2.64	10.46 ± 1.76	< 0.001***
ALT(U/L)	58.09(46.00-69.35)	57.48(45.33–68.83)	62 (49.77–74.31)	< 0.01**
AST(U/L)	43.08(34.00-52.41)	42.74(33.83–52.32)	44.55(36.36–53.36)	0.027*
ALT/AST	1.30(1.03–1.59)	1.29(1.03–1.58)	1.32(1.05–1.66)	0.996
TG(mmol/L)	1.8(0.96–2.40)	1.64(0.90–2.33)	2.43(2.01–2.66)	< 0.001***
TC(mmol/L)	5.05 ± 1.61	4.88 ± 1.60	6.46 ± 0.79	< 0.01**
HDL(mmol/L)	1.27 ± 0.35	1.48 ± 0.47	1.22 ± 0.40	0.422
LDL(mmol/L)	3.28 ± 1.03	3.27 ± 1.02	3.40 ± 1.11	0.277
FPG(mmol/L)	6.50 ± 2.12	6.36 ± 2.16	7.70 ± 1.34	0.001 ***
UA(µmol/L)	362.76 ± 95.53	362.47 ± 96.67	365.28 ± 85.89	0.479
eGFR(mL/min/1.73 m²)	85.62 ± 19.10	89.81 ± 15.59	51.14 ± 5.67	< 0.001***

*DM* Diabetes mellitusm, *SBP *Systolic Blood Pressure, *DBP *Diastolic Blood Pressure, *BMI *Body Mass Index, *CAP *Controlled Attenuation Parameter, *LSM *Liver Stiffness Measurement, *ALT *Alanine Aminotransferase, *AST *Aspartate Aminotransferase, *TG *Triglyceride, *TC *Total Cholesterol, *HDL *High-Density Lipoprotein cholesterol, *LDL *Low-Density Lipoprotein cholesterol, *FPG *Fasting Plasma Glucose, *UA *Uric Acid, *eGFR *estimated Glomerular Filtration Rate

*P < 0.05、**P < 0.01、***P < 0.001 Statistically significant difference

### Multivariable logistic regression analysis of factors associated with CKD in MAFLD

Multivariate logistic regression analysis revealed that Age, LSM, TC, and DM were independent relevant factors for CKD in patients with MAFLD. *P* values for ALT, AST, and TG were > 0.05 (not statistically significant) (Table 2).

**Table 2:** Logistic regression analysis of influencing factors of MAFLD-CKD

Variables	Β value	S.E.	Waldχ²	OR	95%CI	*P*
Age	0.090	0.011	70.133	1.094	1.071 ~ 1.118	< 0.001***
LSM	0.188	0.045	17.221	1.207	1.104 ~ 1.319	< 0.001***
ALT	0.014	0.007	4.036	1.014	1.000 ~ 1.029	0.054
AST	0.006	0.008	0.467	1.006	0.989 ~ 1.029	0.494
TC	0.309	0.089	11.824	1.362	1.142 ~ 1.624	< 0.001***
TG	0.108	0.130	0.660	1.114	0.859 ~ 1.445	0.417
DM(1)#	0.911	0.243	16.609	2.488	0.255 ~ 0.776	< 0.001***

#DM (presence = 1, absence = 0), *P < 0.05、**P < 0.01、***P < 0.001 Statistically significant difference

### Performance of individual and combined indicators in identifying MAFLD-CKD

**Fig. 2:**
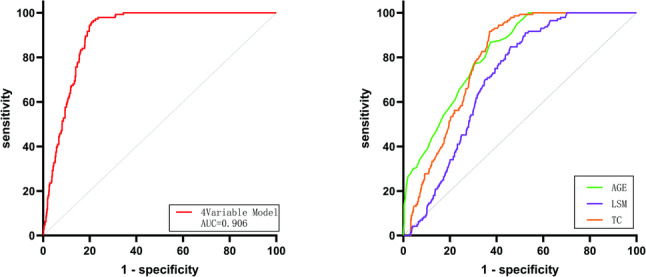
ROC curve analysis of multiple indicators identifying MAFLD-CKD individually and jointly

ROC curve analysis demonstrated that the combined detection of Age, LSM, TC, and the presence of DM for identifying CKD in MAFLD patients yielded an area under the curve (AUC) of 0.899 (95% CI: 0.882 ~ 0.917). This AUC was greater than the corresponding efficacy metrics of any single indicator, and the differences were statistically significant (*P* < 0.05), as shown in Fig. 2. In Table 3, among the individual indicators, Age had the highest specificity, followed by FPG, while Age also showed the highest sensitivity, followed by LSM. The combined detection model exhibited significantly higher AUC values and specificity compared to any single indicator. Based on these findings, the combination of these four variables represents the optimal indicator for the diagnostic efficacy of MAFLD complicated by CKD.Based on the results of the multivariable analysis, a diagnostic model was constructed by incorporating the four identified variables. The model equation is as follows: C = 0.09 × Age + 0.188 × LSM + 0.309× TC + 0.911 × DM (presence = 1, absence = 0) − 11.78.

**Table 3:** Efficacy of multiple indicators and combined detection of MAFLD-CKD

Indicators	Sensitivity	Specificity	AUC	95%CI	*P*
Age	0.65	0.76	0.816	0.786 ~ 0.850	< 0.001***
LSM	0.60	0.66	0.815	0.790 ~ 0.842	< 0.001***
TC	0.49	0.61	0.792	0.765 ~ 0.819	< 0.01**
4 Variable Model	0.71	0.80	0.899	0.882 ~ 0.917	< 0.001***

*P < 0.05、**P < 0.01、***P < 0.001 Statistically significant difference

### Construction of the MAFLD-CKD diagnostic model

**Fig. 3:**
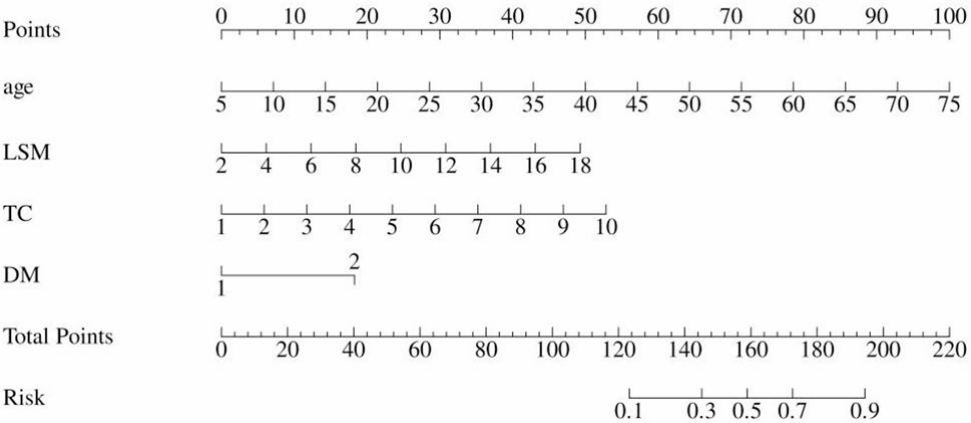
Nomogram of MAFLD-CKD diagnostic model

The aforementioned four variables were incorporated into a diagnostic model. A nomogram was plotted with the outcome variable being the occurrence of MAFLD-CKD. Based on the assigned points for each risk factor on the nomogram, an individual score for each factor can be obtained. The sum of these individual scores corresponds to the probability of an individual developing MAFLD-CKD. The results show that a higher total score is associated with a greater probability of MAFLD-CKD occurrence, as illustrated in Fig. 3.

### Validation of the diagnostic model

**Fig. 4:**
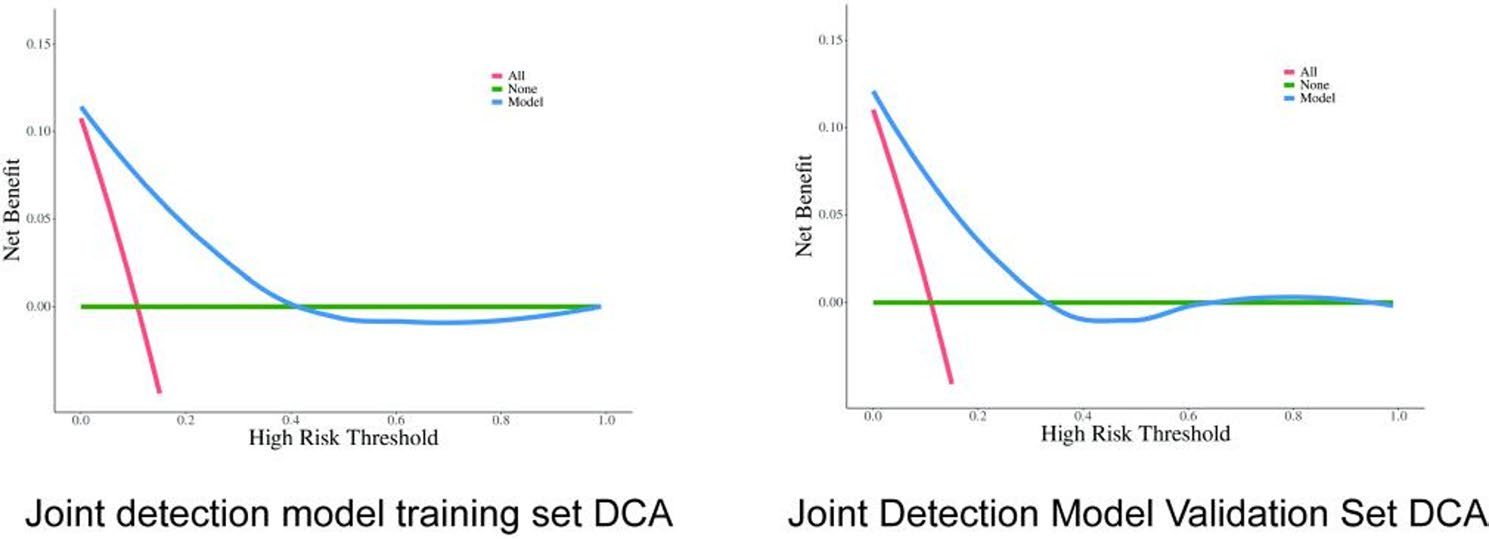
Diagnostic value of MAFLD-CKD in training set and validation set of joint detection model by decision curve analysis

The ROC curve analysis results showed an AUC of 0.91 (95% CI: 0.89 ~ 0.93) for the training set and an AUC of 0.88 (95% CI: 0.85 ~ 0.92) for the validation set (Table 4). These results indicate that the diagnostic model has good discriminative ability in both sample groups. The Hosmer-Lemeshow goodness-of-fit test showed no statistically significant difference between the training and validation set models (*P* > 0.05), meaning the actual probabilities were largely consistent with the model’s identification probabilities, indicating good model calibration. DCA was performed on the combined detection model for both the training and validation sets to assess the model’s net benefit performance across different high-risk thresholds. The results show that the model performs well at lower thresholds, with its advantage gradually diminishing as the threshold increases, as shown in Fig. 4. 

**Table 4:** Diagnostic model confusion matrix: training set + validation set

Data	AUC (95%CI)	Accuracy (95%CI)	Sensitivity (95%CI)	Specificity (95%CI)	PPV (95%CI)	NPV (95%CI)	cut off
Train	0.91 (0.89–0.93)	0.83 (0.80–0.85)	0.81 (0.79–0.84)	0.96 (0.92–1.00)	0.99 (0.99–1.00)	0.38 (0.32–0.44)	0.118
Test	0.88 (0.85–0.92)	0.80 (0.76–0.84)	0.79 (0.75–0.83)	0.89 (0.79–0.98)	0.98 (0.97–1.00)	0.34 (0.26–0.43)	0.118

## Discussion

This study systematically analyzed high-relevant factors for CKD development in MAFLD patients by examining clinical data from 1,328 individuals. The results identified Age, LSM, TC, and the presence of DM as independent relevant factors for MAFLD-CKD, providing a new basis for early kidney risk stratification in MAFLD patients.

Several studies in recent years have explored the relationship between LSM and CKD. For instance, Qin et al. [16] found in a cohort of 1,415 NAFLD patients that LSM was a potential diagnostic indicator for CKD in NAFLD patients. Ciardullo S et al. [17] discovered that LSM was associated with CKD, particularly with the albuminuria phenotype, and this association was independent of traditional relevant factors like obesity and diabetes. Cross-sectional analyses suggest that the risk of developing proteinuria or decreased eGFR is more than double in patients with liver fibrosis compared to the general population, indicating a comorbid mechanism in the pathological progression of the liver and kidneys. These findings align with the identification of LSM as an independent risk factor in this study, further validating the clinical value of LSM in identifying CKD. However, the limitations of LSM should not be overlooked. Its high technical dependency and susceptibility to operator variability necessitate standardized training for healthcare personnel to minimize technical errors in LSM.

Furthermore, a pathogenic link exists between dyslipidemia and CKD. A large-scale study based on the Chinese population found that dyslipidemia in NAFLD patients was linearly and positively correlated with CKD risk and significantly associated with renal function decline [18]. In the analysis by Miao et al. [19], a reduction in TC levels was significantly associated with a decreased risk of CKD, suggesting a potential causal role of lipid metabolism abnormalities in the comorbid mechanism of MAFLD-CKD. This is consistent with our finding that TC level is an independent risk factor for CKD development in MAFLD patients. The association between dyslipidemia and CKD may be attributed to elevated TC levels leading to increased circulating free fatty acids (FFA), which deposit in the kidneys, mediating the activation of lipid peroxidation and mitochondrial dysfunction, thereby inducing podocyte apoptosis and eGFR decline [20]. Although the independent diagnostic value of TC is clear, it is easily influenced by short-term dietary and pharmacological interventions. By constructing a combined diagnostic model, this study not only verified the independent diagnostic value of TC for CKD but also provided a visual risk assessment tool for lipid metabolism management in MAFLD patients from a clinical perspective. The model integrates multiple lines of evidence, significantly improving the identification rate of high-risk individuals.

Diabetes likely serves as a key driver in the progression from MAFLD to CKD. MAFLD patients often exhibit insulin resistance, leading to lipid metabolism disorders that further exacerbate hepatic fat accumulation [21]. Under chronic hyperglycemic conditions, increased formation of advanced glycation end products (AGEs) induces oxidative stress and inflammatory responses in liver cells. Pro-inflammatory factors such as TNF-α, IL-6, ROS, and others drive renal injury and accelerate renal function decline [22]. MAFLD patients with diabetes experience various hormonal imbalances, such as activation of the sympathetic nervous system, water-sodium retention, and abnormal levels of natriuretic peptides due to resistance, leading to impaired renal hemodynamics. This makes insulin resistance a potential mechanistic link between MAFLD and CKD [23].

Currently, the underlying mechanisms of MAFLD-associated CKD remain unclear. Studies have shown that MAFLD and CKD share multiple common metabolic relevant factors. MAFLD, particularly its advanced form MASH, may further increase the risk of developing CKD [24]. Yunpeng Li et al. provided important evidence for understanding the role of hyperuricemia in the pathogenesis of MAFLD by examining the relationship between cumulative uric acid exposure and the incidence of MAFLD [25]. As MAFLD continues to emerge as the most prevalent chronic liver disease worldwide, in-depth research into its relevant factors, especially the impact of long-term metabolic burden, is crucial for developing more effective prevention and intervention strategies [26]. In patients with MASH, hepatocyte injury leads to the release of pro-inflammatory factors and oxidative stress products, which disseminate through the bloodstream to the kidneys, activating intrarenal inflammatory signaling pathways and resulting in glomerular endothelial cell injury and renal tubular epithelial cell apoptosis [27]. Additionally, liver fibrosis is often accompanied by aggravated insulin resistance, which induces metabolic abnormalities and affects the function of renal tubular interstitial cells via the renin-angiotensin system (RAS), inhibiting the accumulation of M2 macrophages and thereby exacerbating renal fibrosis and functional impairment [28].

As a non-invasive and convenient risk diagnostic tool, the nomogram has been widely used in clinical diagnostic research across various fields, including metabolic diseases, oncological diseases, and postoperative complications [29–31]. Developing and validating nomogram-based risk diagnostic models for MAFLD is essential for early screening and management in general populations. These models integrate multiple clinical and lifestyle indicators to provide individualized risk assessment, which helps formulate targeted interventions and reduce the incidence of MAFLD and its related complications, such as cirrhosis, hepatocellular carcinoma, and cardiovascular diseases [32–33]. The MAFLD-CKD risk diagnostic model constructed in this study offers the advantages of operational simplicity and visual interpretability, providing a reliable tool for identifying early-stage MAFLD-CKD patients. ROC curve analysis showed that the AUC values for the training and validation sets were 0.91 (95% CI: 0.89–0.93) and 0.88 (95% CI: 0.85–0.92), respectively, indicating stable discriminatory ability of the model. The Hosmer-Lemeshow goodness-of-fit test further confirmed no statistically significant difference in calibration between the two sets (*P* > 0.05), suggesting good agreement between identified and observed risks. Future research could further explore the application of more advanced machine learning algorithms and deep learning techniques to improve the accuracy and clinical utility of diagnostic models for MAFLD and its related complications [34–35].

In conclusion, this study developed a combined diagnostic model for CKD development in MAFLD by integrating Age, LSM, TC, and the presence of DM. This model demonstrates high diagnostic efficacy and provides an accurate early screening tool for clinical practice, holding significant clinical application value. It offers new insights for the identification, prevention, and control of MAFLD-CKD. Exploring the interaction between LSM and lipid metabolism indicators and their impact on the liver-kidney axis may contribute to a deeper understanding of the pathophysiological processes underlying CKD development in MAFLD patients. Future intervention trials targeting dyslipidemia to assess its renoprotective effects in MAFLD patients could help clarify causal relationships and provide guidance for clinical practice.This study has certain limitations, such as a relatively limited sample size and a lack of long-term follow-up data, which prevents the establishment of dynamic relationships between changes in LSM and TC levels and CKD progression. Future research should aim to address these limitations to enhance the reliability of the findings.

## Conclusion

Age, LSM, TC, and diabetes mellitus were identified as independent relevant factors for CKD in patients with MAFLD. The nomogram model constructed based on these factors demonstrated high identifying accuracy and clinical utility, offering reliable support for renal risk management in MAFLD patients.

### Study strengths and limitations

This integrated model significantly improves the ability to identify the risk of CKD in patients with MAFLD, providing an important tool for early screening and timely intervention. However, this study has several limitations. As a single-center, cross-sectional study with a limited sample size (1,328 cases), its design precludes causal inference. Furthermore, the model lacks external validation, and its generalizability remains to be confirmed. Additionally, it did not incorporate key renal indicators such as the ACR, and liver fibrosis assessment is subject to operator dependence.

Future research should involve multicenter prospective cohort studies and external validation, integrating multidimensional data with technologies such as machine learning to further verify and optimize the model’s clinical predictive value.

## Data Availability

The datasets used are available from the corresponding author upon reasonable request.

## Abbreviations

MAFLD: Metabolic dysfunction-associated fatty liver disease
CKD: Chronic kidney disease
TE: Transient elastography
LSM: Liver stiffness measurement
CAP: Controlled attenuation parameter
SBP: Systolic Blood Pressure
DBP: Diastolic Blood Pressure
DM: Diabetes mellitus
BMI: Body mass index
ALT: Alanine aminotransferase
AST: Aspartate aminotransferase
TG: Triglycerides
TC: Total cholesterol
HDL: High-density lipoprotein cholesterol
LDL: Low-density lipoprotein cholesterol
FPG: Fasting plasma glucose
UA: Uric acid
AUC: Area under the curve
ROC: Receiver operating characteristic
TNF-α: Tumor necrosis factor-alpha
IL-6: Interleukin-6
ROS: Reactive oxygen species
RAS: Renin-angiotensin system
